# Dietary Natural Products for Prevention and Treatment of Liver Cancer

**DOI:** 10.3390/nu8030156

**Published:** 2016-03-10

**Authors:** Yue Zhou, Ya Li, Tong Zhou, Jie Zheng, Sha Li, Hua-Bin Li

**Affiliations:** 1 Guangdong Provincial Key Laboratory of Food, Nutrition and Health, School of Public Health, Sun Yat-Sen University, Guangzhou 510080, China; zhouyuesysu@163.com (Y.Z.); saferide@126.com (Y.L.); zwky740359@163.com (T.Z.); zhengj37@mail2.sysu.edu.cn (J.Z.); 2 South China Sea Bioresource Exploitation and Utilization Collaborative Innovation Center, Sun Yat-Sen University, Guangzhou 510006, China; 3 School of Chinese Medicine, The University of Hong Kong, Hong Kong, China; lishasl0308@163.com

**Keywords:** liver cancer, fruit, vegetable, spice, anticancer

## Abstract

Liver cancer is the most common malignancy of the digestive system with high death rate. Accumulating evidences suggests that many dietary natural products are potential sources for prevention and treatment of liver cancer, such as grapes, black currant, plum, pomegranate, cruciferous vegetables, French beans, tomatoes, asparagus, garlic, turmeric, ginger, soy, rice bran, and some edible macro-fungi. These dietary natural products and their active components could affect the development and progression of liver cancer in various ways, such as inhibiting tumor cell growth and metastasis, protecting against liver carcinogens, immunomodulating and enhancing effects of chemotherapeutic drugs. This review summarizes the potential prevention and treatment activities of dietary natural products and their major bioactive constituents on liver cancer, and discusses possible mechanisms of action.

## 1. Introduction

Globally, liver cancer is the second most common cause of cancer death, accounting for more than 700,000 deaths every year [1,2]. Hepatocellular carcinoma (HCC) is the major type of liver cancer (70%–80%), followed by intrahepatic cholangiocarcinoma [3]. The main risk factors for liver cancer are hepatitis B/hepatitis C virus infection, alcohol consumption, aflatoxin B1 and metabolic disorders [4]. Liver cancer is usually an aggressive malignancy associated with poor prognosis, and the five-year survival rate is estimated to be less than 9%. Surgical interventions including liver resection, liver transplantation and percutaneous ablation are regarded as the most effective approach with curative potential for liver cancer. Unfortunately, due to numerous lesions, and extrahepatic metastasis, only about 20% of liver cancer patients are suitable for surgery. On the other hand, chemotherapeutic drugs for liver cancer are limited, and Sorafenib is the most common prescription. The large phase III trials demonstrated that Sorafenib could improve overall survival and time to progression [5,6]. However, its clinical benefits remains modest, and it was reported that Sorafenib was useful for around 30% patients, and drug resistance developed within six months [7]. Furthermore, problems such as hepatotoxicity, recurrence, drug resistance and other adverse effects exist in current therapeutics, which urge researchers to find alternative treatment. 

Diet plays a pivotal role in cancers. Epidemiological studies suggested that decreased overall cancer risks might be correlated with regular intake of a high fiber, low fat diet accompanied by significant consumption of fruits and vegetables [8,9]. Therefore, dietary natural products could provide novel and fascinating preventive or therapeutic options for liver cancer. Researchers have found a variety of anticancer effects of dietary natural products, such as inhibiting tumor cell growth and metastasis, protecting against liver carcinogens, immunomodulating and enhancing effects of chemotherapeutic drugs [10,11,12]. Furthermore, many dietary natural products displayed selective inhibition against cancer cells [13]. This discrimination is very important for liver cancer treatment, since the majority of patients suffers from severely compromised liver function or liver cirrhosis and can not afford further losses of normal liver cells [4]. This review summarizes the prevention and treatment action of dietary natural products and their major bioactive constituents on liver cancer, and discusses the mechanism of action.

## 2. Fruits

The high content of polyphenols gives fruits remarkable antioxidant activity and may help lessen the risk of cancer [14,15,16]. Indeed, many fruits and their major bioactive constituents showed anticancer potential in various bioassay systems and animal models.

### 2.1. Grape

Grape products are well recognized healthy dietary components against many pathophysiologic processes. Stilbenes, anthocyanins, and procyanidins, which are abundant in grape skin, seeds and red wines, have been reported to possess strong antioxidant and anti-inflammatory properties [17]. A team isolated two fractions (TP-4 and TP-6) from grape cell culture with strong chemopreventive properties in an *in vitro* human DNA topoisomerase II assay [18]. TP-6, the procyanidin-rich fraction, selectively inhibited cell viability of HepG2 cancer cells, yet caused no toxicity to normal PK15 pig kidney cells [13]. Liver cancer is enriched with blood vessels, and angiogenesis plays a key role in cancer metastasis and relapse. The treatment of grape procyanidin in a liver cancer xenograft model exerted anti-angiogenic activity in a dose dependent manner by suppressing proliferation of vascular endothelial cells [19]. Researchers also suggested a possible anti-carcinogenic use against HCC for grape seed extracts from winery waste. The seed extract treatment in HepG2 cells induced DNA damage, enhanced NO production, p53 upregulation and significant decrease of total PARP expression, thus promoting apoptosis [20]. 

### 2.2. Black Currant

Black currant (*Ribes nigrum* L.) fruits are widely consumed, and are known to possess strong antioxidant and anti-inflammatory activities due to high content of anthocyanins (250 mg/100 g fresh fruit), which have been suggested to have potent anti-tumor properties. Utilizing HepG2 cells, an *in vitro* study found that the anthocyanin-rich fraction of black currant significantly inhibited cell proliferation [21]. Compared with other parts, black currant skin extract (BCSE) was a better source of anthocyanins with cyanidin-3-*O*-rutinoside as the predominant one. In a chemically induced rat model of liver cancer, administration of dietary BCSE (100 or 500 mg/kg for 22 weeks) dose dependently suppressed diethylnitrosamine triggered liver γ-glutamyl transpeptidase-positive preneoplastic foci. BCSE also alleviated lipid peroxidation and expression of cyclooxygenase-2, heat shock proteins (HSP70 and HSP90), inducible nitric oxide synthase and 3-nitrotyrosine as well as upregulated many hepatic antioxidant and detoxifying enzymes including glutathione *S*-transferase, quinone oxidoreductase and uridine diphosphate glucuronosyltransferase isoenzymes. The mechanistic study offered substantial evidence that the inhibition of inflammatory cascade via modulating the NF-κB signaling pathway, and suppression of oxidative stress through activating Nrf2 signaling pathway could contribute to the preventive properties of black currant bioactive components against diethylnitrosamine induced hepatocarcinogenesis [10,22]. Similarly, in a diethylnitrosamine initiated and phenobarbital promoted two stage liver cancer rat model, BCSE reduced the incidence, total number, size and multiplicity of preneoplastic hepatic nodules in a dose responsive manner. Further study revealed that the pro-apoptosis via up-regulation of Bax and simultaneously down-regulation of Bcl-2 expression are probably implicated in BCSE-mediated anticancer effects [23].

### 2.3. Plum

Immature plum (*Prunus salicina* Lindl.) fruits contain high contents of natural phenolic phytochemicals, which may be effective dietary natural antioxidants and preventive agents of cancer [24]. The total polyphenol content was 10 g/kg dry weight in extracts of immature plum, and (−)-epicatechin (34.7%) and (−)-gallocatechin gallate (28.6%) were major components. Extract of immature plum induced extrinsic apoptosis in HepG2 cells as evidenced by caspase-8, -10, and -3 activation as well as DNA fragmentation [25]. Another *in vitro* study found anti-metastasis property of immature plum extract in HepG2 cells. Mechanistic analysis suggested that the effects were achieved through inhibition of transcriptional expression of MMP-9 gene via suppressing the nuclear translocations of NF-κB and AP-1 [11]. In liver metabolism of exogenous compounds, phase I reactions are often associated with the metabolic activation of carcinogens, while phase II reactions mediate the detoxify process by facilitating elimination. A team found that pretreatment of immature plum extracts in rat alleviated the carcinogenicity of benzo(α)pyrene (B(α)P) through upregulating the synthesis of enzymes implicated in detoxification [26].

### 2.4. Other Fruits

Pomegranate is gaining increasing attention for its potent antioxidant activities due to rich polyphenol contents, such as anthocyanins, hydrolysable tannins and proanthocyanidins. It was reported that pomegranate bioactive constituents were capable of suppressing diethylnitrosamine induced hepatocarcinogenesis in rats by suppressing oxidative insult through Nrf2-mediated redox signaling pathway and inhibiting inflammatory response via NF-κB-regulated inflammatory pathway. The result provided ample support for potent tumor inhibitory activities of pomegranate at an early stage of hepatocarcinogenesis [27,28].

In China, drinking jujube tea was believed to provide synergic health effects from jujube and tea. Researchers investigated the combined effects of jujube and green tea extract and the underlying mechanisms in an *in vitro* study using HepG2 cells. The combined treatment selectively inhibited cell viability of HepG2 cells, without apparent toxic effects on the normal rat hepatocytes. Furthermore, the combination caused G_1_ cell cycle arrest, which might be associated with increased level of p53 and p21^Waf1/Cip1^ and decreased cyclin E levels. The treatment also enhanced anti-tumor effects via downregulating A proliferation inducing ligand (APRIL), a member of the TNF family which was reported to promote cancer cell growth [29,30].

Apples are well known healthy foods. The flavonoid-enriched fraction isolated from apple peels, at dose of 50 μg total monomeric polyphenols/mL, selectively inhibited cell proliferation of HepG2 cells, which was comparable to the currently prescribed drug Sorafenib. At the same time, the extract showed very low toxicity to normal liver cells. The induction of apoptosis, G_2_/M cell cycle arrest and inhibition of DNA topoisomerase II were suggested to underlie these anti-tumor activities [31].

Sugar apple or sweetsop (*Annona squamosa* L.) is native to the tropical American. The seed extract of the fruit was traditionally used as a remedy against “malignant sores” (cancer) in China. The studies have demonstrated that annonaceous acetogenins, possessing potent anticancer activities, are the major bioactive components of *A. squamosa* seeds. In a recent study, the seed extract showed significant cytotoxicity against HepG2 cells with an IC_50_ of 0.36 μg/mL. The subsequent study in a H22 liver cancer cells transplanted rat model corroborated the findings of *in vitro* study. Oral administration of *A. squamosa* seed extract inhibited the growth of tumor cells with a maximum inhibitory rate of 69.55%. In addition, no adverse effects were observed in response to the extract [32].

Sea Buckthorn (*Hippophae rhamnoides* L.) is a thorny deciduous herb native to Europe and Asia. The fruits are tasty and contain rich nutrients and bioactive substances. The flavonoids of *H. rhamnoides* have been reported to possess antioxidant, immunomodulatory and hepatoprotective activities [33]. Isorhamnetin is an important flavonol aglycone isolated from the plant. *In vitro* study showed that isorhamnetin inhibited cell viability (IC_50_, 74.4 ± 1.13 μg/mL) of BEL-7402 human HCC cells in a time- and dose- dependent manner. The treatment also induced the appearance of a hypodiploid peak, which might due to the promotion of apoptosis [34]. Gac fruit (*Momordica cochinchinensis*) is a delicious wild fruit widely consumed in the Southeast Asia. An *in vitro* study showed that the water extract of Gac fruit exerted antiproliferative activities in HepG2 cells. Immunoblotting found that the treatment downregulated cyclin A, Cdk2, p27^waf1/Kip1^, which might explain the induced S phase arrest. Researchers suggested that a water-soluble protein with molecular weight of 35 kDa was responsible for the anticancer properties [35]. Mangosteen is a common tropical fruit. The fruit hulls contain many xanthones such as α-, β- and γ-mangostin which have diverse biological activities. The antiproliferative activity of xanthones from mangosteen has been demonstrated in several human cancer cell lines from brain, breast and colon. A recent study demonstrated that γ-mangostin had antiproliferative activity in HepG2 cells via induction of apoptosis [36]. Litchi fruit pericarp extract inhibited cancer cell growth (IC_50_, 80 μg/mL) and colony formation *in vitro*. In murine hepatoma bearing-mice, the daily administration of the extract also suppressed tumor growth in a dose-dependent way, with 0.6 g/kg/day inhibited 44.23% (*p* < 0.01) tumor growth [37]. Auraptene is an antioxidant from citrus fruit. Post-treatment of auraptene to *N*,*N*-diethylnitrosamine challenged rats effectively inhibited tumor progression, presumably by negative selection for cancer cells with β-catenin mutation [38]. In an *in vivo* study using Swiss albino mice, both treatments of mango pulp extract and lupeol, a triterpene present in mango, ameliorated DMBA induced alterations in liver [39]. Another study reported that 3,5,7,3′,4′-pentahydroxy-flavonol-3-*O*-β-d-glucopyranoside, ursolic acid and quercetin, which were isolated from cranberry, demonstrated potent antiproliferative effects against HepG2 cells [40]. 

## 3. Vegetables

Epidemiological studies suggested a favorable role of high consumption vegetables, such as cruciferous vegetables, tomatoes and legumes, in cancer risks, particularly of the digestive tract. According to a recent meta-analysis, the intake of vegetables was inversely associated with risk of liver cancer (RR, 0.78; 95% CI, 0.62–0.99) [41].

### 3.1. Cruciferous Vegetables

**Figure 1:**
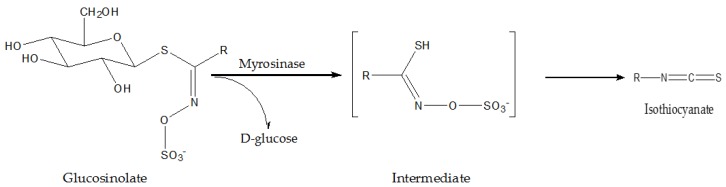
The general structure of glucosinolates and their enzymatic conversion to isothiocyanates by myrosinase.

Many species from the Cruciferae family are widely cultivated and consumed vegetables. Epidemiological studies pointed out consumption of cruciferous vegetables, such as cauliflower, broccoli, watercress and Brussel sprouts, with low risks of various cancers. This anticancer effect has been attributed to high contents of glucosinolates and isothiocyanates in cruciferous vegetables (Figure 1). Radishes (*Raphanus sativus* L.) contained high concentrations of glucosinolates, isothiocyanates and polyphenols [42]. For instance, 4-methylsulfanyl-3-butenyl glucosinolate, also referred to as glucoraphasatin, is a glucosinolate which is most abundant in *Raphanus sativus*. It has been well accepted that an effective way for achieving anti-tumor activities is facilitating phase II detoxification enzyme systems, such as NAD(P)H: quinone oxidoreductase 1 (NQO1), glutathione-*S*-transferase and phenolsulfotransferases, thereby promoting the detoxification of reactive metabolites of carcinogenic compounds [43]. Indeed, numerous studies have demonstrated the anti-tumor effects of glucosinolates which were associated with promotion of such enzyme activities [44]. However, it was suggested that isothiocyanate, rather than glucosinolate itself, possessed anticarcinogenic activity. For instance, in precision-cut rat liver slices, low concentrations of glucoraphasatin as well as its corresponding isothiocyanate derived from radish sprouts potently upregulated hepatic phase II detoxification enzymes involved in the metabolism of chemical carcinogens, including mycotoxins, heterocyclic amines and polycyclic aromatic hydrocarbons, while it left the cytochrome P450 enzymes such as the CYP1 family unaffected [45]. Furthermore, sulforaphane (SUL), an isothiocyanate particularly high in broccoli, was reported to transcriptionally increase the expression of CYP1A1 in a time- and dose-responsive fashion in Hepa 1c1c7 and HepG2 cells [46]. However, it was suggested that SUL was highly reactive and was further metabolized to the *N*-acetyl-l-cysteine (NAC) conjugate in humans. The SUL-NAC showed greater effects of inhibition against murine hepatoma cells and induction of activities of quinone reductase, a phase II detoxification enzyme [47]. Sinigrin, a main aliphatic glucosinolate present in cruciferous vegetables, is hydrolyzed to allylisothiocyanate (AITC). Both treatments of AITC and synthetic NAC-AITC dose dependently inhibited the growth of Hepa1c1c7 murine hepatoma cells. The increased activity and mRNA expression of quinine reductase might be responsible for the observed anticancer effects [48]. Besides, in SK-Hep 1 human hepatoma cells, the treatment of the two compounds dose dependently suppressed cancer cell adhesion, invasion, and migration through downregulating matrix metalloproteinase (MMP)-2/-9 at a transcriptional level [49]. Rutabaga (*Brassica napobrassica*) is a popular vegetable in North Europe and North America. Extract of rutabaga (especially eight day sprouts) exerted selective antiproliferative and pro-apoptotic effects in HepG2 cells, while it had less potent effects on the growth of normal mammalian Chinese hamster ovary cells [50].

### 3.2. French Bean

A study evaluated the antiproliferative activities of aqueous extracts from aerial parts of French bean (*Phaseolus vulgaris*). The extracts at 400 and 800 mg/mL displayed potent antioxidant activities, and also suppressed the growth of HepG2 cells by 57% and 74%, respectively [51]. Phytochemical analysis of the seed coats from *P. vulgaris* identified 24 compounds, including 12 triterpenoids and seven flavonoids. Several compounds exhibited antiproliferative activities with IC_50_ ranging from 32.1 ± 6.3 to 779.3 ± 37.4 ìM [52]. Legume lectins are usually the abundant storage proteins in legumes. In recent years, lectin has received special attention as therapeutic agents due to its diverse biological functions including anti-tumor, antibacterial and anti-HIV activities. A dimeric 64-kDa hemagglutinin was isolated from dried seeds of *P. vulgaris* with a high yield (1.1 g/100 g seeds). The compound displayed a modest inhibition against the growth of HepG2 cells (IC_50_, 100 μM), without interfering in normal liver WRL 68 cells [53]. Later, the team purified a new legume lectin (BTKL) from seeds of *P. vulgaris*, which possessed strong selectively cytotoxicity to HepG2 cells (IC_50_, 7.9 ± 0.5 μM). According to their study, the potential mechanisms of the anti-tumor activities of BTKL include: (1) inducing apoptosis and necrosis; (2) promoting NO production via the upregulation of iNOS; and (3) triggering the release of pro-inflammatory cytokines such as IL-1β, IL-2, TNF-α, and INF-γ [54]. In another study, a hemagglutinin isolated from *P. vulgaris* showed stronger antiproliferative properties than concanavalin A in the HepG2 cancer cell line [55].

### 3.3. Tomato

It was reported that tomato contains an average of 11.6–14 mg/kg lycopene. The compound is an unsaturated carotenoid with high antioxidant activity, which has been reported to modulate cell proliferation, differentiation, and apoptosis [56,57]. Therefore, lycopene might serve as a rational approach in combating HCC. The pre-treatment of lycopene from tomatoes to *N*-nitrosamines challenged mice ameliorated the carcinogenic damage, as shown by decrease of oxidative stress, chromosomal and membrane abnormalities [58]. Moreover, lycopene administration markedly suppressed the expression of anti-apoptotic gene and upregulated caspase 3, 9 and p53 expression, leading to enhanced apoptosis in response to *N*-nitrosamine insult [59]. Besides, in *N*-nitrosodiethylamine (NDEA)-challenged mice, decrease of tumor incidence (42.05%), multiplicity (3.42), and burden (1.39) as well as increase of survival rate were observed upon lycopene pretreatment to NDEA-treated animals. Histopathological analysis showed a reduction of aggressive tumor nodules formation [60]. Collectively, these results may support anti-tumor properties of lycopene during early stages of chemical induced hepatocarcinogenesis.

Beside lycopene, tomatine, a glycoalkaloid contained much higher in green tomato than the red one, may also possess anti-tumor properties. *In vivo* study indicated that anti-tumor effects of tomatine acted through a different mechanism, including induction of antigen-specific cellular immunity and direct destruction of cancer cell membranes. A commercial tomato glycoalkaloid tomatine (10:1 mixture of α-tomatine and dehydrotomatine) exerted dose-dependent inhibition against HepG2 cancer cells and was reported to be more potent than the anticancer drug doxorubicin. These findings suggested that consumers might also benefit from eating high-tomatine containing green tomatoes [61].

### 3.4. Asparagus

Asparagus (*Asparagus officinalis* L.) is a popular vegetable often used in soups, salads and vegetable dishes. Several studies revealed numerous pharmacological activities associated with *A. officinalis*, such as anti-inflammation, anti-mutagenicity, and cytotoxicity. Polysaccharides, steroidal saponins and flavonoids extracted from the plant were suggested to be main constituents responsible for its bioactivities.

Asparagus polysaccharide has been clinically adopted to treat various cancers including breast cancer, leukemia, and lung cancer. A recent study reported that the asparagus polysaccharide selectively inhibited cell proliferation of HepG2 (IC_50_, 5.7 mg/mL) and Hep3B (IC_50_, 9.39 mg/mL) cell lines with less toxicity on normal human hepatocellular 7702 cells (IC_50_, 20.92 mg/mL). Mechanistic study revealed that the induction of G_2_/M phase arrest and apoptosis by asparagus polysaccharide via modulation of Bax, Bcl-2 and capase-3 contributed to the effects [62]. In addition, asparagus polysaccharide was a good embolic candidate in transcatheter arterial chemoembolization (TACE), a minimally invasive treatment for unresectable HCC. The combined treatment of asparagus polysaccharide with TACE markedly suppressed liver tumor growth as well as prolonged survival time in rat model with little toxicity [63].

Asparanin A, a steroidal saponin isolated from *A. officinalis*, has displayed antiproliferative activities against many cancers, such as esophageal cancer, gastric cancer, lung cancer and leukemia [64]. Asparanin A also exerted dose- and time-dependent inhibition against HepG2 cells with IC_50_ at 6.20 ± 0.56 μmol/L. The treatment induced G_2_/M cell cycle arrest through downregulating Cdk1, Cdk4, and cyclin A and simultaneously upregulating p21^WAF1/Cip1^. Besides, the promotion of apoptosis via both the intrinsic and extrinsic pathway was observed upon asparanin A treatment to HepG2 cells [65]. 

### 3.5. Other Vegetables

The treatment of mung bean sprouts (MBS) extract showed different cytotoxicity against HepG2 cells (IC_50_, 14.04 ± 1.5 mg/mL) and normal human cells (IC_50_, 163.97 ± 5.73 mg/mL). The selectivity index for HepG2 cells was 11.94 ± 1.2. Mechanisms underlying the anti-tumor properties of MBS included induction of apoptosis (Bax and capase-8), increase of anti-tumor cytokines (TNF-α and IFN-β), promotion of IFN-γ production, and upregulation of cell-mediated immunity through immunopolarization [66].

*Momordica charantia* lectin (MCL) is a type II ribosome inactivating protein derived from bitter gourd, a vegetable and traditional herbal medicine in China. The treatment of MCL significantly suppressed HCC cell growth *in vitro* and *in vivo* through inducing G_2_/M phase arrest, apoptosis and autophagy [67]. Besides, MAP30, a type I ribosome inactivating protein purified from bitter gourd, demonstrated both cytostatic and cytotoxic effects in cultured Hep G2 cells. The activities were attributed to activation of extrinsic and intrinsic caspase apoptosis and induction of S phase cell cycle arrest. The anti-tumor role of MAP30 was also demonstrated *in vivo* [68]. RNase MC2 is a ribonuclease from *M. charantia*, and could enhance apoptotic death both *in vitro* and in HepG2-bearing mice [69].

*Perilla frutescens* L. is widely used for its pleasant aroma and the leaves are eaten as a delicious vegetable. Ingredients extracted from the plant, including rosmarinic acid, caffeic acid and apigenin were reported to exert antiproliferative activities against a wide range of cancers. A study reported that isoegomaketone, a compound isolated from *P. frutescens*, significantly inhibited cell growth and xenograft tumor formation probably through blocking the PI3K/Akt signaling pathway of HCC cells [70]. A study evaluated the antioxidant activity, contents of total phenolics, anthocyanin and chlorogenic acid, and *in vitro* anticancer capacity of potato. Among the tested samples, *Solanum pinnatisectum*, which possessed the highest antioxidant activity, also showed the strongest antiproliferative effects against liver cancer cells [71]. It was suggested that the glycoalkaloids from potatoes possessed anti-tumor abilities. The treatment of potato glycoalkaloids, especially α-chaconine dose dependently inhibited HepG2 cell growth in the range of 0.1–10 μg/mL, with lower cytotoxicity to normal liver cells [72]. Celery (*Apium graveolens* L.) is frequently used as vegetable worldwide. Its seeds, possessing potent antioxidant and anti-inflammatory abilities, are traditionally used to treat liver indurations. Phytochemical analysis reported that main bioactive constituents in celery seeds were apigenin, linamarose, and vitamins A and C. Pretreatment of rats with celery seed extracts dose dependently suppressed chemically induced hepatocarcinogenesis as evidenced by reduction of γ-GT positive foci [73].

## 4. Spices

### 4.1. Garlic

**Figure 2:**
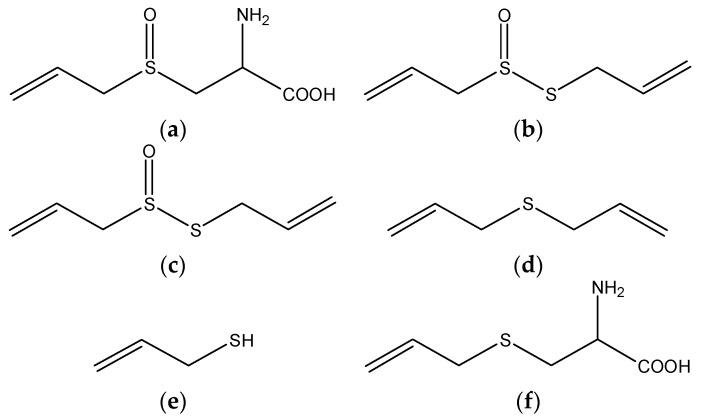
The chemical structures of several organo-sulphur compounds in garlic: (**a**) alliin; (**b**) allicin; (**c**) diallyl disulfide; (**d**) diallyl sulfide; (**e**) allyl mercaptan; and (**f**) *S*-allylcysteine.

Epidemiologic evidence suggested that high consumption of garlic protected against various cancers. Organo-sulphur compounds (OSC), such as alliin, allicin, diallyl disulfide, diallyl sulfide, allyl mercaptan, and *S*-allylcysteine, were reported to be major ingredients with anti-tumor properties in garlic (Figure 2) [74,75]. For instance, administration of garlic powders to rat inhibited DNA damage by 35%–60% induced by *N*-nitrosodimethylamine in liver as assessed by the comet assay. The effect was attributed to the high alliin concentration in samples [76]. Subsequently, researchers investigated the anticancer effects of selected OSC from garlic against chemical induced DNA damage using HepG2 cells. The study showed that all the OSC tested except allyl mercaptan markedly inhibited aflatoxin B1 induced DNA damage, while allyl mercaptan administration significantly reduced DNA breaks induced by dimethylnitrosamine. Benzo(α)pyrene genotoxicity was effectively suppressed by diallyl disulfide. Besides, all the tested OSC inhibited DNA damage of direct-acting agents, H_2_O_2_ and methyl methanesulfonate [77]. In another study using rat hepatoma cells, sodium 2-propenyl thiosulfate was found to be a potent inducer of quinone reductase [78]. In Hep3B HCC cells, hexane extracts of garlic promoted ROS production and subsequent dysregulation of mitochondrial membrane potential, leading to enhanced apoptotic cell death [79]. Similarly, allicin was also able to induce apoptotic cell death through overproduction of ROS in Hep3B human HCC cell line [80]. The propensity to metastasis of HCC leads to recurrence and poor prognosis. *S*-allylcysteine was observed to suppress proliferation and metastasis of HCC in a metastatic HCC cell line MHCC97L and *in vivo* xenograft liver cancer model. The potential mechanisms included (1) to inhibit cancer cell migration and invasion by suppressing VEGF and increasing E-cadherin; (2) to promote cell apoptotic death via downregulating Bcl-2,-xl and upregulating caspase-3, -9 activities; and (3) to induce S cell cycle arrest [79]. On the other hand, an *in vitro* study reported that the water-soluble garlic extracts were more potent inhibitor of HepG2 cells than the oil-soluble compound diallyl disulfide by inducing a p53/p21-mediated G_2_/M phase arrest and apoptosis [81]. A team evaluated the anti-tumor activities of a unique garlic preparation, namely aged garlic extract in rats. The preparation inhibited diethylnitrosamine induced preneoplastic lesions in liver, presumably through slowing the proliferation rate of liver cells [82]. It is known that immune functions are usually deficient in advanced-cancer patients. A clinical trial of patients with advanced cancer in digestive system (84% were liver cancer) reported that the aged garlic extract had a positive effect on natural-killer cell activities [83].

### 4.2. Turmeric

Turmeric (*Curcuma longa* L.) is a popular spice in Asia, especially in India. In HBV X protein transgenic mice, *Curcuma longa* extracts led to less visceral fat, lower ratio of liver to body weight and delayed pathogenesis. Since HBV infection plays a key role in the development and progression of liver cirrhosis and HCC, the spice may be a good candidate against HBV-related liver cancer [84]. Curcumin is a yellow pigment from *Curcuma longa* with numerous bioactivities including antioxidant, anti-inflammatory and anticancer activities. In an *in vivo* study, curcumin demonstrated anticancer activity against chemical induced hepatocarcinogenesis. The administration of curcumin reduced hyper plastic nodule, liver damage markers, body weight loss and hypoproteinemia in the liver of diethylnitrosamine/phenobarbital challenged Wistar rats [85]. The protective effects of curcumin against liver cancer also involved the enhanced degradation of hypoxia-inducible factor, and curcumin could promote apoptosis of Hep 3B cells [86]. It was suggested that curcumin glucuronide was the main form of curcumin in plasma following oral administration in rats, because most curcumin was conjugated after absorption. This conjugated compound showed weaker anticancer activities against HepG2 cancer cells [87]. Beside curcumin, curcuma oil possessed hepatoprotective properties, and sesquiterpenoids might be its main bioactive ingredients. Treatment of curcuma oil alleviated concanavalin A induced oxidative stress and inflammation in mice. The oil also induced apoptosis of Hepa1-6 cancer cells in a time-/dose-dependent manner *in vitro* and inhibited inoculated tumor cell growth *in vivo* [88]. Aromatic tumerone (ar-tumerone) is another volatile oil from *C. longa*. The IC_50_ of ar-tumerone were 64.8 ± 7.1 μg/mL for HepG2, 102.5 ± 11.5 μg/mL for Huh-7, and 122.2 ± 7.6 μg/mL for Hep3B liver cancer cell line. Further analysis showed that the ar-turmerone-induced apoptotic cell death of HepG2 was a result of ROS-mediated ERK and JNK kinases activation [89].

### 4.3. Pepper

An *in vitro* study showed that pepper extracts reduced cell viability of rat hepatoma McA-RH7777, while displaying no cytotoxicity, even protected against basal death of normal rat hepatocytes in the case of *Piper putumayoense*. The selective cytotoxicity was attributed to the intracellular accumulation of ROS [90].

The glycoprotein (24 kD) isolated from *Zanthoxylum piperitum* could prevent chemical-induced liver carcinogenesis by immunomodulation and promotion of apoptosis. In diethylnitrosamine treated Balb/c mice, the glycoprotein (20 mg/kg) enhanced expression of perforin, granzyme B and NK cell activities, as well as pro-apoptotic factors (bid, capase-3 and cytochrome c) in liver [91]. The metastasis of tumor required degradation of extracellular matrix. MMP-2/-9 promoted this process, while TIMP-1/-2, the endogenous inhibitors of MMPs, was negatively related to tumor metastasis. *In* *vitro* studies using HA22T cancer cell line, treatment of *Zanthoxylum avicennae* extract not only suppressed cell proliferation through induction of G_2_/M cell cycle arrest and apoptosis, but also inhibited cell metastasis, invasion via downregulating MMP-2/-9 and upregulating TIMP-1/-2 [92,93,94]. Subsequent mechanistic analysis revealed that the activation of phosphatase 2A was behind those anti-tumor effects. An essential oil was extracted from dried pericarp of *Zanthoxylum schinifolium*, which mainly consisted of 29.9% geranyl acetate, 15.8% citronella and 15.4% sabinene. This volatile extract dose dependently promoted ROS production in HepG2 cells, leading to increased apoptotic cell death. The extract also exerted anti-tumor activities in Huh-7 human liver cancer cell transplanted nude mice [95].

### 4.4. Ginger

It was reported that ginger could be a promising candidate for cancer prevention [96,97,98]. In a chemical induced liver cancer rat model, 50 mg/kg daily treatment of ginger significantly reduced serum liver cancer markers (α-fetoprotein, CEA), as well as liver tissue growth factors [99]. The inhibition of inflammation and promotion of apoptosis were implicated in the protection of ginger against liver cancer. For instance, the suppression of inflammatory responses as evidenced by decreased NF-κB and TNF-α was found in ginger (100 mg/kg) treated rat hepatoma model [100]. Besides, ginger extracts dose dependently inhibited cell proliferation of the HEp-2 cell line (IC_50_, 900 μg/mL), which was mediated though ROS induced apoptotic death. Further analysis of active ingredients by GC-MS revealed the existence of geraniol, pinostrobin and clavatol [101]. In addition, the studies suggested that 6-shogaol and 6-gingerol, two compounds isolated from ginger, exhibited anti-metastasis effects against liver cancer cells via downregulation of MMP-9, urokinase-type plasminogen (6-shogaol) and upregulation of TIMP-1 [102]. Moreover, 6-shogaol could also effectively induce ROS-mediated caspase-dependent apoptosis in a multidrug resistance hepatoma cell line [103].

### 4.5. Other Spices

Star anise (*Illicium verum*) is widely consumed as a condiment in Asian countries. In an NDEA/phenobarbital induced liver cancer model, the administration of star anise during the promotion stage exhibited anti-carcinogenic potential in the liver tissue of rat. The treatment ameliorated tumor burden (decrease of liver weight, nodule incidence, size, volume and multiplicity), decreased oxidative stress (restoration of superoxide dismutase activity) and upregulated phase II detoxifying enzymes (glutathione-*S*-transferase) [104].

Saffron is the dried stigmas of *Crocus sativus* L., which are commonly consumed as spice and food colorant. Saffron has been proposed as a potential treatment for cancer. The IC_50_ of saffron against HepG2 cells was 950 μg/mL. The inhibition of cancer cell viability by saffron involved apoptosis, but was not associated with ROS production [105]. The induction of apoptosis was also observed in saffron treated rats after diethylnitrosamine administration. Besides, saffron reduced tumor burden and oxidative damage as well as suppressed inflammatory responses in the liver tissue [106].

Galangal (*Alpinia officinarum* H.) is a spice in southern China. Galagin, a flavonol from *A. Officinarum*, could promote apoptotic death of HCC cells in the intrinsic mitochondrial pathway via activation of capase-8 and Bid [107]. The diarylheptanoids isolated from the roots also possessed modest cytotoxicity against HepG2 liver cancer cells [108]. Isoobtusilactone A is isolated from *Cinnamomum kotoense* leave. The compound could induce apoptotic cell death (IC_50_, 37.5 μmol/L) through overproduction of ROS in HepG2 cells [109]. In addition, *in vitro* studies showed that the ROS-mediated anticancer effect was also involved in the promotion of TRAIL-related apoptosis by isoobtusilactone A [110]. A study showed that basil extract could inhibit the sulfotransferase induced procarcinogenesis by suppressing DNA adducts formation in HepG2 cells and in rat hepatoma model [111]. In addition, carnosic acid from rosemary exerted anti-tumor activities against aflatoxin B1 through reduced oxidative stress in HepG2 cells [112].

## 5. Soy

The decreased cancer risks of Asian population traditionally eating a soy-based diet have been associated with the abundant soy isoflavones with antioxidant activities, especially daidzein and genistein [113]. For instance, the treatment of daidzein at a non-toxic dose enhanced the activity and transcriptionally upregulated the expression of catalase in Huh-7 and HepG2 human liver cancer cells [114]. In another study, a trypsin inhibitor isolated from Hokkaido large black soybeans exerted antiproliferative (IC_50_, 140 μmol/L) activities against HepG2 cells [115]. Similarly, a trypsin inhibitor (19 kD) from Chinese black soybean *Glycine max* inhibited growth of HepG2 cells with an IC_50_ of about 25 μmol/L [116]. However, it is of note that the role of soy isoflavones as food supplements in cancer prevention is controversial. It was reported that genistein possessed abilities to promote proliferation of estrogen-dependent breast cancer *in vivo*, which could be ameliorated by other components in soy foods [117]. Thus, the consumption of soy-based foods rather than purified soy isoflavones might be safer for women at high risk of breast cancer.

## 6. Cereals

Rice bran is a byproduct of rice milling. In a study, gastrointestinal-resistant peptide hydrolysates prepared from rice bran were fractionated into different size. Trypan blue dye exclusion assay showed that the <5, 5–10 kD fractions significantly suppressed (*p* < 0.05) growth of HegG2 cells *in vitro* [118]. The byproduct is also a source of phytic acid [119], which has been reported to possess anticancer abilities. Phytic acid from rice bran could dose-dependently suppress HepG2 cancer cell proliferation with an IC_50_ of 2.49 mmol/L. The growth inhibition was correlated to enhanced apoptosis as shown by upregulated capase-3/-9, Bax and p53 [120]. Pigmented rice usually contained more bioactive compounds, such as flavones, phenolics, tannin, tocopherols and sterols [121]. For example, Payao, a pigmented rice cultivar from Thailand, was reported to be a rich source of anthocyanin (5.80 mg/g). The extract of Payao could significantly inhibit HepG2 cell growth [122].

Corn is a widely cultivated economic crop. Polysaccharides from corn silk exerted anti-tumor effects and extended survival time of H22 hepatoma-bearing mice. The enhanced immune function, as evidenced by increase of IL-2/-6, and TNF-α, peripheral white blood cells counts, thymus and spleen index following the polysaccharides treatment, might delineate these anti-tumor activities [123]. *Coix lacryma*, also called semen coicis, is a grass-like relative of corn in China. Extract from the seed of *Coix lacryma* time- and dose-dependently promoted apoptotic death of HepG2 cancer cells through upregulation of capase-8 [124]. According to an *in vitro* study, the ethyl acetate and hexane extracts of buckwheat hull selectively inhibited cell growth by 75.3% and 83.6% of Hep3B liver cancer cells, while the inhibition rates against normal control cells were lower than 35% [125].

## 7. Edible Macro-Fungi

Edible macro-fungi have a long history of use for its nutrition value and flavors. It is also a rich source of bioactive compounds, especially polysaccharide [126]. Recently, the exploration of edible macro-fungi for tumor prevention and treatment has been carried out in various model systems.

*Agaricus blazei* M. has been suggested to have anticancer activities. There was evidence that the mushroom could improve immune function and life quality of gynecological cancer patients taking chemotherapy [127]. Besides, the extract from *A. blazei* meycelial could decrease formation of abnormal collagen fiber in HCC cells [128], which might be very helpful since liver cirrhosis is highly implicated in the development of liver cancer. The anti-tumor property of *A. blazei* was also proved in Smmu 7721 hepatoma-bearing mice [129]. Further studies isolated several compounds with anti-tumor activities against liver cancer. For instance, the β-glucan from *A. blazei* protected against benzo(á)pyrene induced DNA damage in HepG2 cells by binding to the carcinogen, scavenging free radicals and probably modulating cell metabolism [130]. Two compounds, namely blazeispirols A and C from *A. blazei* displayed potent antiproliferative activities against Hep3B cells (IC_50_, 2.8 and 4.5 μg/mL) and HepG2 cells (IC_50_, 1.4 and 2.0 μg/mL) [131]. Mechanistic study reported that bazeispirol A inhibited Hep3B cancer cell growth in a dose- and time-dependent manner through promoting apoptotic death [132].

*Pleurotus pulmonarius* had potent antioxidant activities. Pretreatment of *P. pulmonarius* to Huh7 hepatoma bearing-nude mice significantly reduced the incidence and size of tumors without obvious adverse effects [133]. Furthermore, *P. Pulmonarius* could inhibit invasion and drug-resistance of hepatoma cells. The inhibition of the PI3K/AKT signaling pathway was suggested to be the underlying mechanism [134]. In another study, protein isolated from the dried fruiting bodies of *Pleurotus eryngii* dose dependently suppressed HepG2 cell proliferation through apoptosis without apparent toxicity to normal liver Chang cells [135]. A polysaccharide (120 kDa) from *Pleurotus abalones* also possessed antiproliferative properties in HepG2 cells [136]. In addition, the polysaccharide-rich fraction of *Lentinula edodes* mycelia effectively killed HepG2 cells through the capase-3/-8 mediated extrinsic apoptosis pathway, but showed minor cytotoxicity on normal control cells [137]. 

AAL-2 is a novel lectin (43.175 kDa) from *Agrocybe aegerita*. *In vitro* study reported that the lectin could bind to the surface of liver cancer cells, resulting in apoptotic cell death. Furthermore, AAL-2 administration inhibited tumor growth and extended survival time in hepatoma-bearing mice [138]. The study reported a glycoprotein (FVE) with immunomodulatory properties from *Flammulina velutipes* [139]. In hepatoma-bearing mice, the oral treatment of FVE (10 mg/kg) significantly prolonged survival time and reduced tumor size, through inducing cytotoxic immune response by enhancing innate and adaptive immunity. Besides, IFN-γ was suggested to participate in this process [140]. Iso-suillin, isolated from *Suillus luteus*, selectively suppressed proliferation, induced G_1_ cell cycle arrest and promoted apoptotic death of SMMC-7721 human liver cancer cells, without obvious cytotoxicity against normal human lymphocytes [141]. *O*-orsellinaldehyde was isolated from *Grifola frondosa*. The compound exhibited selective potent cytotoxicity against Hep3B cells (IC_50_, 3.6 μg/mL) through apoptosis [142]. In addition, *p*-terphenyl derivatives from *Thelephora aurantiotincta* induced G_1_ phase arrest in human hepatoma cells probably through iron chelation [143].

## 8. Effects of Combination of Dietary Natural Products with Anticancer Treatments

Chemotherapy and radiotherapy are commonly used cancer therapies. However, the toxic adverse effects and drug resistance restrict their clinical effectiveness. Dietary natural products and their bioactive components could improve efficacy, decrease dose, and ameliorate toxic effects of anticancer drugs, and the combination of natural products with anticancer treatments could offer an attractive strategy for liver cancer treatment. Resistance to apoptosis is a common trait of many cancer cells, which has become a hurdle in traditional anticancer therapies. Asparagus polysaccharide could potentiate the tumoricidal activities of mitomycin *in vitro* and *in vivo*, reducing the amount of drug used without causing deleterious effects. The promotion of apoptosis was suggested to contribute to the activities [62]. In another study, *Momordica charantia* lectin enhanced Sorafenib induced apoptosis by 3.37 folds in HepG2 cells. Consistently with the *in vitro* finding, the combined treatment at a physiologically safe dose completely arrested HepG2 xenograft tumor growth [67]. Extracts of Payao (pigment rice) also sensitized HepG2 cells to cytotoxicity of vinblastine, which was suggested to be achieved through a mitochondrial apoptosis pathway [122]. In addition, enhancing anticancer activities through promotion of intracellular drug accumulation was observed in several studies. For instance, compared with doxorubicin alone, the combination of grape proanthocyanidin and doxorubicin markedly inhibited H22 tumor growth. The promotion of doxorubicin-induced apoptosis via intracellular doxorubicin accumulation is likely to be the underlying mechanism [144]. Epigallocatechin gallate also induced intracellular accumulation of doxorubicin in a human chemoresistant liver cancer cell line through suppressing the activity of a P-glycoprotein efflux pump [145]. NF-κB regulates cell survival and its activation contributes to drug resistance via inhibiting the pro-apoptotic effects of chemotherapy. The studies demonstrated that *Agaricus blazei* and *Hericium erinaceus* could sensitize doxorubicin-mediated apoptosis through inhibiting NF-κB activation [146,147]. It was reported that the PI3K/AKT pathway contributed to drug resistance of cancer cells, and inactivation of the pathway by *Pleurotus pulmonarius* significantly enhanced the sensitivity of HCC cells to cisplatin [134]. In addition, the destruction of lymphocytes and immunosuppression were problems associated with anticancer therapies. A study showed that combined treatment of polysaccharides from *Lentinus edodes* and *Tricholoma matsutake* enhanced anticancer activities of 5-fluorouracil against H22 cells. Besides, compared with 5-fluorouracil alone, the combination performed better in reducing tumor weight and volume in mice model. It was suggested that the enhanced activities of NK cells and cytotoxic T lymphocytes, the increased secretion of cytokines (TNF-α and IFN-γ) and frequency of CD4^+^ and CD8^+^ T cells in the spleen as well as maintainence of the relative weight of the thymus and spleen all contributed to chemo-sensitizing activities of *Lentinus edodes* and *Tricholoma matsutake* [12]. On the other hand, liver is considered to be sensitive to radiation, and radiotherapy itself could induce liver damage. Compared with radiotherapy alone, the combination of apricot supplementation and radiotherapy exerted synergistically protective effects against DMBA (7,12-dimethylbenz(a)anthracene) induced liver damage and carcinogenesis in rats through alleviating apoptosis and oxidative stress [148].

**Figure 3:**
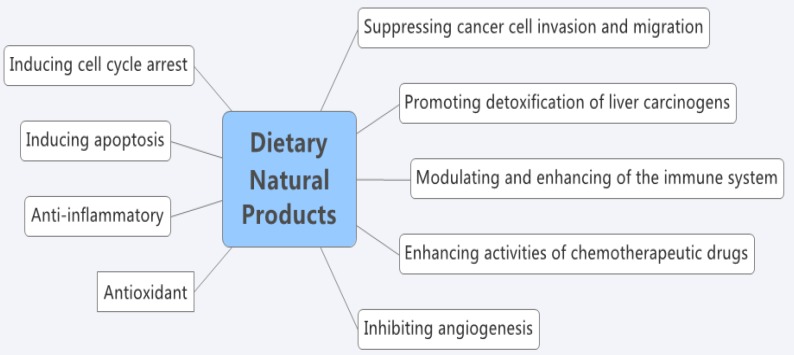
Some effects of dietary natural products against liver cancer.

Finally, some dietary natural products for the prevention and treatment of liver cancer are summarized in Table 1. In addition, some effects of dietary natural products against liver cancer are shown in Figure 3.

## 9. Conclusions

Accumulating evidence suggested that many dietary natural products could be potential sources for prevention and treatment of liver cancer, and the following are notable for their potential anti-hepatoma properties, including (1) grapes, black currant, plum, pomegranate, and the isolated flavonoids, tannins, proanthocyanidins; (2) cruciferous vegetables (isothiocyanates), French beans (lectins), tomatoes (lycopene and tomatine), asparagus (polysaccharides and saponins); (3) garlic (organo-sulphur compounds), turmeric (curcumin), ginger (6-shogaol and 6-gingerol); and (4) soy, rice bran, and polysaccharides from edible macro-fungi. These dietary natural products and their active components could affect the development and progression of liver cancer in various ways, such as inhibiting tumor cell growth and metastasis, protecting against liver carcinogens, immunomodulating and enhancing effects of chemotherapeutic drugs. In the future, attention should be paid to the isolation of active compounds, the illustration of action mechanisms, bioavailability, potential toxicity and adverse effects, and more studies are required concerning the clinical efficacy of dietary natural products and their bioactive components.

## Figures and Tables

**Table 1:** Effects of dietary natural products against liver cancer.

Natural Products	Bioactive Components	Study Type	Bioactivities and Potential Mechanisms	References
Fruits				
Grape	procyanidins	*in vitro*	selective cytotoxicity to cancer cells	[13]
		*in vivo*	inhibiting tumor angiogenesis; promoting doxorubicin induced apoptosis	[19,144]
	Flavan-3-ol	*in vitro*	inducing apoptosis, DNA damage and Suppressing expression of oncoprotein Her-2	[20]
Black currant	anthocyanins	*in vivo*	protecting against diethylnitrosamine induced hepatocarcinogenesis by inducting apoptosis and suppressing oxidative stress and inflammation	[10]
Plum	polyphenols	*in vitro*	inducing extrinsic apoptosis and inhibiting migration	[11,25]
		*in vivo*	protecting against B(α)P liver damage through regulating enzymes involved detoxification	[26]
Pomegranate	polyphenols	*in vivo*	protecting against diethylnitrosamine induced hepatocarcinogenesis by suppressing oxidative stress and inflammatory responses	[27,28]
Apple	polyphenols	*in vitro*	inducing apoptosis, G2/M cell cycle arrest and inhibiting DNA topoisomerase II in cancer cells	[31]
Sweetsop	annonaceous acetogenins	*in vitro* and *in vivo*	exerting cytotoxicity against HepG2 cells and inhibiting tumor growth in hepatoma bearing mice	[32]
Sea buckthorn	isorhamnetin	*in vitro*	promoting apoptosis of human hepatoma cells	[34]
Gac fruit	a water soluble protein	*in vitro*	inducing S phase arrest in cancer cells	[35]
Mangosteen	γ-mangostin	*in vitro*	inducing apoptosis in cancer cells	[36]
Citrus fruit	auraptene	*in vivo*	suppressing tumor progression in *N*,*N*-diethylnitrosamine challenged rats by negative selection for cancer cells with β-catenin mutation	[38]
Mango	lupeol	*in vivo*	ameliorating DMBA insult induced alterations in liver	[39]
Vegetables				
Radish	Glucoraphasa-tin, isothiocyanate	*in vitro*	upregulating hepatic phase II detoxification enzymes involved in the metabolism of chemical carcinogens	[45]
Broccoli	sulforaphane	*in vitro*	upregulating CYP1A1 and quinone reductase	[46,47]
Rutabaga	NA	*in vitro*	exerting selective antiproliferative and pro-apoptotic effects in cancer cells	[50]
French bean	triterpenoids, flavonoids	*in vitro*	exhibiting antiproliferative activities against cancer cells	[52]
	lectins	*in vitro*	exerting selectively cytotoxicity to cancer cells via promoting apoptosis, necrosis, NO production and release of proinflammatory cytokines	[54]
Tomato	lycopene	*in vivo*	protecting against chemical induced liver carcinogenesis through inducing apoptosis	[59,60]
	tomatine	*in vivo*	inducing antigen-specific cellular immunity and direct destructing cancer cell membranes	[61]
Asparagus	polysaccharides	*in vitro* and *in vivo*	selectively inhibiting cancer cell proliferation and enhancing the tumoricidal activities of mitomycin	[62]
		*in vivo*	serving as a good embolic candidate in transcatheter arterial chemoembolization	[63]
	asparanin A	*in vitro*	inducing G2/M cell cycle arrest and apoptosis	[65]
Mung bean sprouts	NA	*in vitro*	increasing apoptosis, anti-tumor cytokines (TNF-α and IFN-β), IFN-γ production and upregulating cell-mediated immunity	[66]
Bitter gourd	lectin	*in vitro*	inducing G2/M phase arrest, apoptosis, autophagy and enhancing the anti-tumor effects of Sorafenib	[67]
	MAP30	*in vitro* and *in vivo*	inducing apoptosis and S phase cell cycle arrest	[68]
Purple perilla	Isoegomake-tone	*in vitro* and *in vivo*	inhibiting cell growth and xenograft tumor formation probably through blocking the PI3K/Akt signaling pathway	[70]
Potato	glycoalkaloids	*in vitro*	selectively inhibiting cancer cell growth	[72]
Celery	pigenin, linamarose, Vitamins A/C	*in vivo*	dose dependently suppressing chemically induced hepatocarcinogenesis	[73]
Spices				
Garlic	Organo-sulphur compounds	*in vitro*	inhibiting chemical induced DNA damage	[77]
	sodium 2-propenyl thiosulfate	*in vitro*	upregulating quinone reductase	[78]
	allicin	*in vitro*	inducing apoptosis through overproduction of ROS	[80]
	S-allylcysteine	*in vitro* and *in vivo*	inducing apoptosis and S phase arrest, inhibiting cancer cell migration and invasion	[78]
	aged garlic extract	*in vivo*	inhibiting diethylnitrosamine induced preneoplastic lesions in liver	[81]
		clinical trial	enhancing natural-killer cell activities	[83]
Turmeric	NA	*in vivo*	protecting against HBV-related liver cancer	[84]
	curcumin	*in vitro* and *in vivo*	demonstrating anti-tumor activity against chemical induced hepatocarcinogenesis	[85]
	Sesquiterpe-noids	*in vitro* and *in vivo*	alleviating concanavalin A induced oxidative stress and inflammation, inhibiting cancer cell growth	[88]
	aromatic tumerone	*in vitro*	inducing apoptotic cell death via ROS-mediated ERK and JNK kinases activation	[89]
Pepper	NA	*in vitro*	selective cytotoxicity against rat hepatoma cells through intracellular accumulation of ROS	[90]
	glycoprotein	*in vivo*	preventing chemical induced liver carcinogenesis by immunomodulation and promotion of apoptosis	[91]
	NA	*in vitro*	inducing G2/M cell cycle arrest and apoptosis, inhibiting cell metastasis, invasion via down-regulating MMP-2/-9 and up-regulating TIMP-1/-2	[92,93,94]
	geranyl acetate, citronella, sabinene	*in vitro* and *in vivo*	increasing apoptotic cell death through ROS production	[95]
Ginger	NA	*in vivo*	inhibiting inflammation and promoting apoptosis	[100]
	geraniol, pinostrobin, clavatol	*in vitro*	inhibiting cancer cell proliferation though ROS-mediated apoptotic death	[101]
	6-shogaol, 6-gingerol	*in vitro*	suppressing metastasis via down-regulation of MMP-9, urokinase-type plasminogen and up-regulation of TIMP-1	[102]
Star anise	NA	*in vivo*	ameliorating tumor burden, oxidative stress and upregulating phase II detoxifying enzymes	[104]
Saffron	NA	*in vitro* and *in vivo*	inducing apoptosis and decreasing tumor burden, oxidative damage and inflammatory responses	[105,106]
Galangal	galagin	*in vitro*	promoting mitochondrial apoptotic death	[107]
Cinnamon	Isoobtusilac-tone A	*in vitro*	inducing apoptotic cancer cell death through overproduction of ROS	[109,110]
Basil	NA	*in vitro* and *in vivo*	inhibiting sulfotransferase induced procarcinogenesis by suppressing DNA adducts formation	[111]
Rosemary	carnosic acid	*in vitro*	protecting against aflatoxin B1 through reduced oxidative stress	[112]
Soy	trypsin inhibitor	*in vitro*	inhibiting cancer cell growth	[115,116]
Cereals				
Rice bran	peptide hydrolysates, phytic acid	*in vitro*	inhibiting cancer cell growth	[118,120]
Pigmented rice	anthocyanin	*in vitro*	synergistically promoting the cytotoxicity of vinblastine through a mitochondrial apoptosis pathway	[122]
Corn silk	polysaccharides	*in vivo*	enhancing immune function and extending survival time	[123]
Semen coicis	NA	*in vitro*	dose-dependently promoting apoptotic death of cancer cells through upregulation of capase-8	[124]
Edible macro-fungi				
*Agaricus blazei*	NA	*in vitro*	sensitizing doxorubicin induced apoptotic death of cancer cells; decreasing formation of abnormal collagen fiber	[128,146]
	β-glucan	*in vitro*	protecting against B(α)P induced DNA damage by binding to the carcinogen, scavenging free radicals and probably modulating cell metabolism	[130]
	blazeispirols A and C	*in vitro*	displaying potent antiproliferative activities against Hep3B cells and HepG2 cells	[131]
*Pleurotus pulmonarius*	NA	*in vitro* and *in vivo*	reducing the incidence and size of tumor; inhibiting invasion and drug-resistance of hepatoma cells; enhancing cytotoxicity of cisplatin	[133,134]
*Lentinula edodes*	polysaccharide	*in vitro*	selectively killing HepG2 cells through the capase-3/-8 mediated extrinsic apoptosis pathway	[137]
*Agrocybe aegerita*	lectin	*in vitro*	binding to the surface of liver cancer cells, resulting apoptotic cell death	[138]
*Flammulina velutipes*	FVE (glycoprotein)	*in vivo*	prolonging survival time and reduced tumor size through inducing cytotoxic immune response	[140]
*Suillus luteus*	iso-suillin	*in vitro*	selectively inducing G1 cell cycle arrest and apoptotic death in cancer cells	[141]
*Grifola frondosa*	*O*-orsellinaldehyde	*in vitro*	exhibiting selective potent cytotoxicity against Hep3B cells	[142]

NA stands for not available.
